# Electrochemical metallization cell with solid phase tunable Ge_2_Sb_2_Te_5_ electrolyte

**DOI:** 10.1038/s41598-018-29778-9

**Published:** 2018-08-14

**Authors:** Ziyang Zhang, Yaoyuan Wang, Guanghan Wang, Jiaming Mu, Mingyuan Ma, Yuhan He, Rongrong Yang, Huanglong Li

**Affiliations:** 1 Department of Precision Instrument, Center for Brain Inspired Computing Research, Tsinghua University, Beijing, 100084 China; 2 Department of Electronic Engineering, Tsinghua University, Beijing, 100084 China

## Abstract

Electrochemical metallization (ECM) cell kinetics are strongly determined by the electrolyte and can hardly be altered after the cell has been fabricated. Solid-state property tunable electrolytes in response to external stimuli are therefore desirable to introduce additional operational degree of freedom to the ECM cells, enabling novel applications such as multistate memory and reconfigurable computation. In this work, we use Ge_2_Sb_2_Te_5_(GST) as the electrolyte material whose solid state is switched from the amorphous(a) to the crystalline(c) phase thermally. Electrical heating too is readily achievable. The resistive switching characteristics of the cells with different GST phases are examined. The magnitude of the high resistance, the SET voltage and the on/off ratio are found to be considerably affected by the solid phase of GST, whereas the magnitude of the low resistance is least affected. Moreover, a transition from volatile to nonvolatile SET switching is only observed for c-GST based cell under prolonged voltage sweep, but not for a-GST based cell. This work provides a springboard for more studies on the manipulation of the ECM cell kinetics by tunable electrolyte and the resulting unprecedented device functionalities.

## Introduction

The modern semiconductor technologies are rapidly reaching the physical limitations of downscaling electronic elements^1^. To address this challenge, a shift to new physical concepts is essential. ECM memories are among the various emerging nonvolatile memory (NVM) technologies, holding the potential to replace Flash and enable novel memory and computing architectures to circumvent the von Neumann bottleneck^2,3^. Typically, the ECM cells have simple metal–electrolyte–metal structure, where a solid electrolyte layer is sandwiched between an active electrode (AE), e.g., Cu or Ag, and an inert electrode (IE), e.g., Pt, W or Au. The ECM cell can be switched between the high resistance state (HRS) and the low resistance state (LRS). When a positive bias is applied to the AE, the AE is electrochemically oxidized and releases cations which then drift across the electrolyte toward the IE under the electric field, and are subsequently electrochemically reduced and nucleate, leading to the formation of metallic filament within the electrolyte and therefore reducing the resistance. This process is known as the SET of the cell. If then a large enough voltage of the opposite polarity is applied, the reversed electrochemical process occurs to dissolve the filament, switching the cell back to the HRS. Due to the existence of an initial conducting path, the local temperature increases significantly due to the Joule heating effect which facilitates the disconnection of the filament by the diffusion of cations into the surroundings under their concentration gradient^4,5^. This process is known as the RESET of the cell. Since the ECM cells operate on ionic carriers and their resistive switching relies on the ion movement within the electrolyte, they have the particular advantage to mimic biological synapses due to the similar operation principle, and therefore have also aroused great interest from the neuromorphic research community^6^. The ECM cell kinetics has been found to be highly electrolyte dependent, leading to diverse filament growth modes and structures^7^. Various types of materials, such as Cu_2_S, Ag_2_S, GeS_x_, Ta_2_O_5_ and a-Si, have been considered for the electrolytes of ECM cells and their influence on the resistive switching characteristics has been reported^8–14^. Menzel *et al*. reported that for Cu_2_S, Ag_2_S and GeS_x_ electrolytes the switching takes place at very low voltage due to the high initial mobile cation concentration or high cation conductivity but for Ta_2_O_5_ and a-Si electrolytes with low mobile cation concentration the switching occurs at high voltage^15^. Suri *et al*. reported that the Ag/GeS_2_/W ECM cell shows abrupt SET switching, which is desirable to emulate the probabilistic learning of biological synapses^12^. On the other hand, Jo *et al*. reported that the ECM cell utilizing a-Si electrolyte shows more gradual switching, which is suitable for the analog emulation of deterministic synaptic learning^14^. Tsuruoka *et al*. found that the redox current is enhanced and the forming voltage of the Cu/Ta_2_O_5_/Pt cell is reduced when the density of the Ta_2_O_5_ film is decreased^13^.

Synergizing multiple functions into a single device or enabling function tunability is technologically important. This requires the use of tunable material in response to external stimulus. For example, replacing conventional MgO barrier in the magnetic tunnel junction (MTJ) with multiferroic barrier whose electrical polarization can be tuned by the external voltage bias, Gajek *et al*. doubled the storage density of a single MTJ cell^16^. Yoon *et al*. demonstrated that the synaptic plasticity can be selectively activated by modulating the polarization of the ferroelectric electrolyte used in their ECM cell^17^. Phase change materials (PCMs), such as GST, belong to another important class of tunable materials whose solid phases (amorphous and crystalline), and the associated electrical and optical properties can be changed by heating, induced thermally, electrically or optically^18^. Due to the unique properties, PCMs have been widely used in commercialized optical memories and NVMs^19^. Recently, Deleruyelle *et al*. reported the use of a-GST as the electrolyte material in ECM cells and demonstrated typical bipolar switching behavior^20^. However, the impact of different solid phases of GST on the switching characteristics of the ECM cells, which is the fundamental toward enabling multi-functionalities or function tunability of GST based ECM cells, remains unclear. Here, we fabricate the ECM cell with GST electrolyte and study the GST solid phase dependent switching characteristics of the cell. This work provides guidelines for the optimization of ECM cells with tunable GST electrolyte and is the starting point for applications based on the GST introduced operational degree of freedom to the ECM cells.

## Results and Discussion

**Figure 1:**
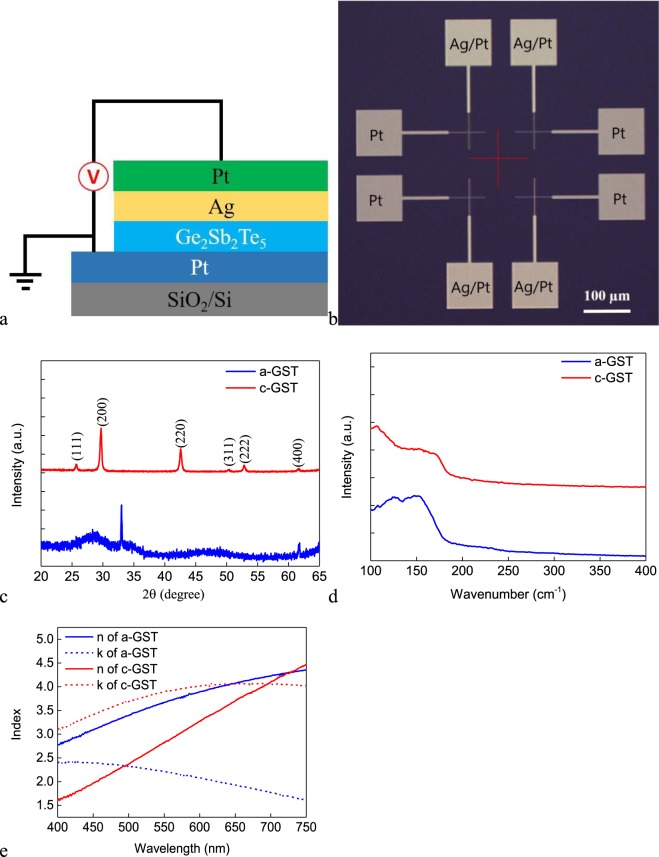
(**a**) The schematic structure of the ECM cell. (**b**) Optical image of the device with 2 × 2 μm^2^ junction area. (**c**) XRD data of the as-deposited and annealed GST films. (**d**) Raman spectra of the as-deposited and annealed GST films. (**e**) Experimentally measured refractive indexes of GST in its amorphous and crystalline states.

The Ag/GST/Pt cell with crossbar electrode structure is fabricated and its schematic structure is shown in Fig. 1a. The optical image of the device with 2 × 2 μm^2^ junction area is shown in Fig. 1b. To examine the structure of the as-deposited GST, XRD and Raman spectroscopy analyses are carried out for a 100 nm-thick GST film deposited on the Si wafer. For XRD, the two weak and broad peaks at around 28 degrees shown in Fig. 1c indicate that our as-deposited GST film is amorphous^21^. Raman spectrum shown in Fig. 1d provides further evidences. The broad peak at around 150 cm^−1^ observed for the as-deposited GST film may be due to the Te–Te stretching mode, which is mostly regarded as a signature of the amorphous phase^22^. To crystallize the GST film, the film is annealed on a hotplate under cleanroom condition. The full crystallization of GST is confirmed by both XRD, as is shown in Fig. 1c and Raman spectroscopy, as is shown in Fig. 1d. For XRD, the GST film annealed at 220 degrees for 15 minutes shows diffraction peaks in the directions of (111), (200), (220), (311), (222) and (400), indicating that the GST film is crystallized in the rock-salt (Fm3m) structure^21^. For Raman spectrum, the single broad peak at 150 cm^−1^ splits into two peaks, the positions of which are below and above 150 cm^−1^, respectively. The lower peak is mostly associated with either GeTe_6_ octahedral or Sb-Te bonds within defective octahedral, which can be regarded as a signature of the crystalline phase^22^. The large optical contrast of GST between its crystalline and amorphous phases in the visible range is measured by the variable-angle spectroscopic ellipsometry (Fig. 1e).

**Figure 2:**
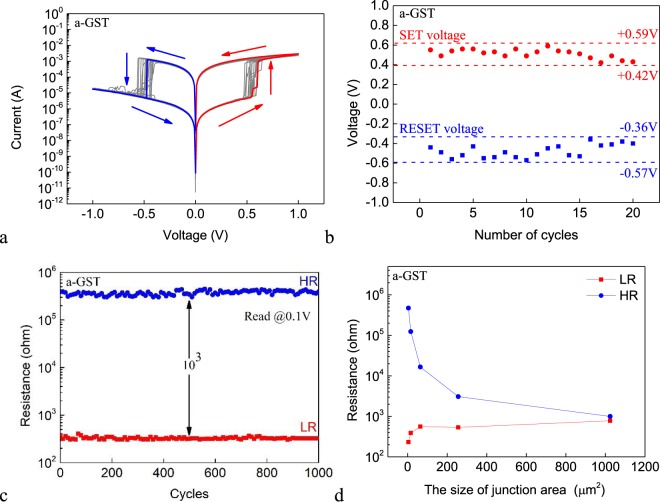
(**a**) The DC I–V curves of 20 consecutive sweep cycles for the Ag/a-GST/Pt cell. (**b**) Statistical distribution of the SET voltage and RESET voltage for the Ag/a-GST/Pt cell. (**c**) The LR and HR of the Ag/a-GST/Pt cell for 1000 consecutive sweep cycles. The voltage of the read pulse is 100 mV. (**d**) The dependence of the LR and HR on the size of the junction area for the Ag/a-GST/Pt cell. Unless otherwise stated, the sweep rate is fixed to be 1 V/s for all measurements in this work.

Figure 2a shows the DC I–V characteristics of 20 consecutive sweep cycles for the Ag/a-GST/Pt cell with 2 × 2 μm^2^ junction area. The pristine cell is in the HRS. The voltage on the Ag AE is first swept in the positive direction. At certain voltage between +0.4 V and +0.6 V, the cell switches to the LRS. When reversing the voltage polarity, the cell switches back to the HRS at certain absolute value of voltage less than 0.7 V. The switching behavior can be understood by the ECM mechanism: when applying a positive voltage to the Ag AE, Ag will be oxidized to Ag^+^ cations which migrate toward the Pt IE, and are subsequently reduced back to Ag and nucleate, forming Ag conductive filament(s) and therefore reducing the cell resistance. Upon reversal of the voltage polarity, the redox reactions are also reversed. In the meantime, Joule heating effect also assists the electro-migration and diffusion of the cations. Both field and thermal effects lead to the dissolution of the conductive filament and therefore restoring the HRS.

Figure 2b shows the statistical distribution of the SET voltage and RESET voltage for the Ag/a-GST/Pt cell from these 20 sweeps. The SET voltage varies in the range between +0.42 V – +0.59 V and the RESET voltage in the range between −0.57 V – −0.36 V. The SET voltage and RESET voltage are non-overlapping, which is essential for memory applications. Figure 2c shows the low resistance (LR) and high resistance (HR) for 1000 sweeps. The resistance ratio between the HRS and the LRS maintains to be larger than 10^3^ during the test. The retention of the HRS and LRS of the device has also been tested (see Fig. S1a,b) up to 8 hours, during which the resistance values are found to be stable. Figure 2d shows the dependence of the LR and HR on the size of the junction area. The HR decreases with increasing size of the junction area, whereas the LR remains almost constant, which is due to the filamentary nature of the resistive switching. Therefore, further reduction of the the size of the junction area is expected to improve the on/off ratio.

**Figure 3:**
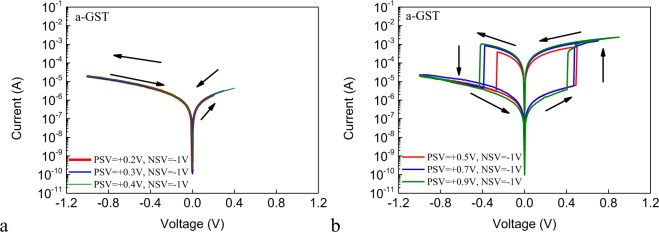
(**a**) The DC I–V curves for low PSVs applied on the Ag/a-GST/Pt cell, the NSVs are fixed to −1 V. (**b**) The DC I–V curves for high PSVs applied on the Ag/a-GST/Pt cell, the NSVs are fixed to −1 V.

Figure 3a and b shows the DC I–V curves of the Ag/a-GST/Pt cell with different positive stop voltages (PSVs). The negative stop voltage (NSV) of each sweep is fixed to −1 V. The size of the junction area of the cell is 2 × 2 μm^2^. The voltage on the Ag AE is first swept in the positive direction. From Fig. 3a, we see that no resistive switching occurs when the PSV is below +0.4 V, around which the SET voltage lies in, indicating that the electrical field is not strong enough to induce the formation of Ag conductive filament(s). Increasing the PSV to above +0.5 V, nonvolatile bipolar resistive switching is found, as is shown in Fig. 3b. This is in accordance with the requirement for voltage above the threshold (SET voltage) to enable the electrocrystallization of Ag at the IE^23^. For the minimum PSV (+0.5 V) considered here that enables bipolar resistive switching, the absolute value of the RESET voltage is found to be lower than those of the other sweeps with higher PSVs, despite of the similar SET voltages. In order to verify if the increase of the RESET voltage with increasing PSV is related to decreasing LR, we measure the LRs of the device obtained under different PSVs, as shown in Fig. S2a. We see that the LRs are of the same order of magnitude and are almost independent of the PSVs. This can alternatively be understood from the I–V hysteresis behavior. As shown in Fig. 3b, voltage sweeping between the SET voltage and the PSV does not induce I–V hysteresis, implying that a PSV larger than the SET voltage no longer induces change of the resistance in a nonvolatile manner. Therefore, we propose another possible reason of the increase of the RESET voltage with increasing PSV. Although the resistance switching in the ECM cells is generally believed to be field driven, Joule heating also plays significant role in facilitating the formation of nanofilaments or introducing second-order effects^24^. In our case, the prolonged voltage sweep with larger PSV will generate more Joule heat that may enhance the motion of the Ge, Sb and Te atoms in the vicinity of the filament. This facilitates the reconstruction of the interface of the embedded Ag nanofilament and the GST matrix toward more stable configuration. As a result, the filaments formed under larger PSVs are stabilized and their rupture requires larger RESET voltage. Meanwhile, since this is an interfacial process, the filamentary conductivity is not much affected. Confirmation of this hypothesis awaits further studies. Noticing that the PSV does not affect the SET resistance significantly but does affect the RESET voltage, we also expect that such properties of PSV could be exploited to enable second-order memristors or to emulate the bio-phenomena that rely on a second state variable, such as meta-plasticity.

We also consider the effects of sweep rate on the SET and RESET voltages, as shown in Fig. S3. It can be seen that the SET voltage decreases exponentially with increasing sweep rate. This is probably due to the facilitated growth of the conducting filaments under high sweep rate, in analogy to the pair-pulse facilitation effect found in high frequency pulse train measurements^24,25^. On the other hand, the RESET voltage does not have monotonic dependence on the sweep rate.

**Figure 4:**
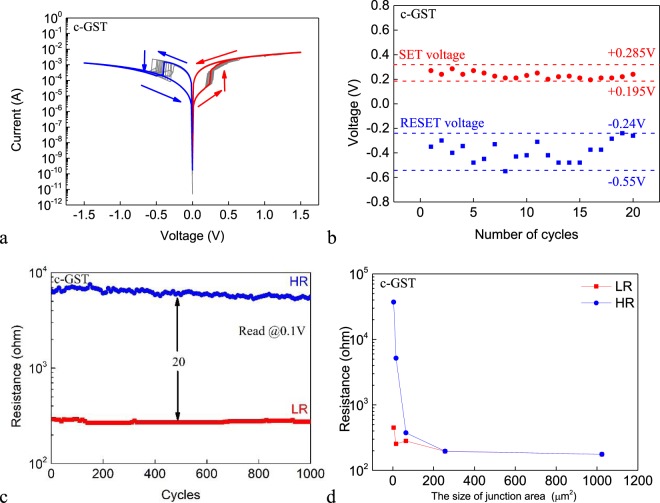
(**a**) The DC I–V curves of 20 consecutive sweep cycles for the Ag/c-GST/Pt cell. (**b**) Statistical distribution of the SET voltage and RESET voltage for the Ag/c-GST/Pt cell. (**c**) The LR and HR of the Ag/c-GST/Pt cell for 1000 consecutive sweep cycles. The voltage of the read pulse is 100 mV. (**d**) The dependence of the LR and HR on the size of the junction area for the Ag/c-GST/Pt cell.

To obtain the Ag/c-GST/Pt cell, the Ag/a-GST/Pt cell is annealed at 220 degree for 15 minutes on a hotplate. Then we measure the electrical properties of the Ag/c-GST/Pt cell to study the GST solid phase dependent switching characteristics. Figure 4a shows the DC I–V characteristics of 20 consecutive sweep cycles for the Ag/c-GST/Pt cell with 2 × 2 μm^2^ junction area. The voltage on the Ag AE is first swept in the positive direction. The cell also shows repeatable bipolar resistive switching behavior.

Figure 4b shows the statistical distribution of the SET voltage and RESET voltage for the Ag/c-GST/Pt cell. The SET voltage varies in the range between +0.195 V – +0.285 V and the RESET voltage in the range between −0.55 V – −0.24 V. The SET voltage and RESET voltage are non-overlapping, which is essential for memory applications. Compared with the Ag/a-GST/Pt cell, the SET voltage of the Ag/c-GST/Pt cell is decreased, which may be related to the different film morphologies of a-GST and c-GST and consequently the different cell kinetics, including cation mobilities and redox rates^15^. The possible Ag related effects induced by annealing are also evaluated (see Fig. S4). It is believed that the phase transition of GST is the key factor to enable device tunability.

Figure 4c shows the LR and HR for 1000 consecutive sweeps. The resistance ratio between the HRS and the LRS maintains to be larger than 20 during the test. Compared with the Ag/a-GST/Pt cell, the LRs for both cells are similar, which can be understood by the formation of Ag conductive filament(s) of similar sizes. On the other hand, the HR of the Ag/c-GST/Pt cell is much lower than that of the Ag/a-GST/Pt cell. This attributes to the bulk conduction instead of filamentary conduction of the HRS and therefore the HR is mainly determined by the bulk resistance of the electrolyte. In the case of GST electrolyte, the crystalline state has much lower resistance than the amorphous state, resulting in lower HR of the Ag/c-GST/Pt cell. The retention of the HRS and LRS of the device has also been tested (see Fig. S1c,d) up to 8 hours, during which the resistance values are found to be stable.

Figure 4d shows the dependence of the LR and HR on the size of the junction area for the Ag/c-GST/Pt cell. The LR is independent of the size of the junction area, remaining almost constant, but the HR increases with decreasing size of the junction area, which is due to the filamentary nature of the resistive switching.

**Figure 5:**
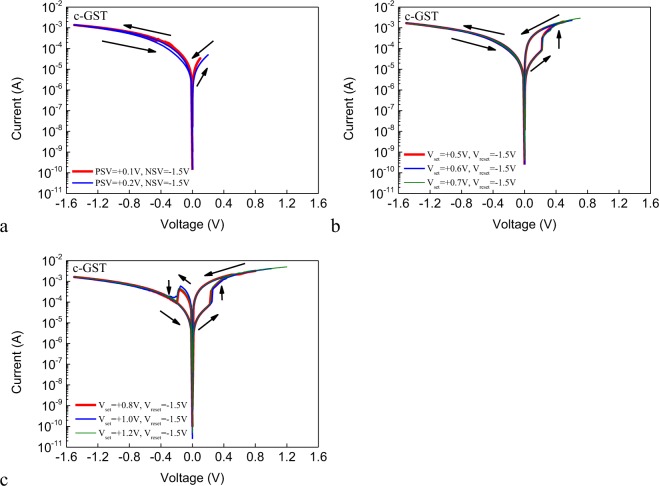
(**a**) The DC I–V curves for low PSVs applied on the Ag/c-GST/Pt cell, the NSVs are fixed to −1.5 V. (**b**) The DC I–V curves for medium PSVs applied on the Ag/c-GST/Pt cell, the NSVs are fixed to −1.5 V. (**c**) The DC I–V curves for high PSVs applied on the Ag/c-GST/Pt cell, the NSVs are fixed to −1.5 V.

Figure 5 shows the DC I–V curves of the Ag/c-GST/Pt cell with different PSVs. The NSV of each sweep is fixed to −1.5 V. The size of the junction area of the cell is 2 × 2 μm^2^. The voltage on the Ag AE is first swept in the positive direction. Under low PSV below +0.3 V, around which the SET voltage lies in, there is no resistive switching behavior, indicating that the electrical field is not strong enough to induce the formation of Ag conductive filament(s). Increasing the PSV to +0.5 V, +0.6 V or +0.7 V, SET switching is observed. During the successive voltage sweep in the negative side, however, the device is found to have returned to the HRS and no switching occurs, indicating the spontaneous rupture of the filament(s) or the volatility of the SET switching. The appearance of volatile switching in c-GST but not in a-GST may be attributed to the different interfacial energies of the Ag filament: GST interfaces formed in GST of different phases^26^. Further increasing the PSV to above +0.8 V, stable bipolar nonvolatile resistive switching occurs, which affirms the importance of PSV or the prolonged voltage sweep in stabilizing the filament(s). As the PSV effect may not be limited to GST, it provides guidelines for controlling the device volatility and the transition to non-volatility, which is highly useful in designing memory devices, selector devices and neuromorphic devices. Moreover, GST serves as a tunable electrolyte which enables additional operational degree of freedom for novel devices with unprecedented functionalities.

## Conclusion

In conclusion, we fabricate the ECM cell with solid phase tunable Ge_2_Sb_2_Te_5_ electrolyte. The utilization of the solid-state property tunable electrolytes in response to external stimuli in our device introduces additional operational degree of freedom to the ECM cells. The electrical properties of the Ag/a-GST/Pt and Ag/c-GST/Pt cell are examined. It is found that the magnitude of the HR, the SET voltage and the on/off ratio is considerably affected by the solid phase of GST, whereas the magnitude of the LR is least affected. In addition, a transition from volatile to nonvolatile resistive switching can be found in the Ag/c-GST/Pt cell only under prolonged voltage sweep. The results not only open an encouraging avenue to manipulating the ECM cell kinetics by tunable electrolyte, but also provide guidelines for designing memory devices, selector devices and neuromorphic devices of novel functionalities.

## Experimental Section

Si (ASM, Netherlands) with 300 nm thermally grown silicon dioxides (SiO_2_) is used as the substrate for the thin film deposition. Prior to the deposition of the thin films, substrates are cleaned in acetone under ultrasonic agitation, then rinsed in isopropanol, and dried under nitrogen flow. All the films are deposited using a magnetic sputtering system (AJA, USA) at room temperature in 20 sccm (standard cubic centimeters per minute) Argon (Ar) atmospheres. 5 nm Ti (adhesion layer) and 20 nm Pt (bottom electrode) are first fabricated by photolithographic patterning followed by magnetron sputtering and lift-off process. Then 40 nm GST (electrolyte layer), 20 nm Ag (top electrode) and 100 nm Pt (antioxidation layer) are fabricated using the same procedure, obtaining different junction areas (2 × 2, 4 × 4, 8 × 8, 16 × 16 and 32 × 32 μm^2^). The as-deposited GST films are amorphous. The cells are annealed on a hotplate under cleanroom condition so as to crystallize the GST.

The electrical characterizations are performed using Keysight B1500A semiconductor cell analyzer, which is equipped with high-resolution source and measurement units to sense the current with an integration time of 16 power line cycles with a specified resolution of 1 fA. All electrical measurements are performed at room temperature and under an ambient atmosphere. The X-Ray Diffraction (XRD) is performed using X-Ray diffractometer (Rigaku, Japan) on 100 nm thick GST (deposited on SiO_2_/Si substrate) with different degrees of crystallization, where the diffraction angle is set from 10° to 90°. For the study of Raman spectra using LabRAM HR (Horiba Jobin Yvon, France), a He-Ne laser (*λ* = 632.8 nm) is used as excitation beam and the power density of the laser beam is smaller than 0.2 mW/μm^2^ which will not induce the crystallization of GST. The complex refractive indexes of GST are measured using a spectroscopic ellipsometer (Ellitop, China) at the light incident angle of 70° with a wavelength scanning step of 1 nm from 400 nm to 750 nm.

## Electronic supplementary material

Supplementary Information
