# Supplementary material for: Electrochemical metallization cell with solid phase tunable Ge2Sb2Te5 electrolyte
Source: Sci Rep. 2018 Aug 14;8:12101. doi: 10.1038/s41598-018-29778-9 (PMC6092410; doi:10.1038/s41598-018-29778-9)
Supplement: Supplementary file 1 — Supplementary Information [file 41598_2018_29778_MOESM1_ESM.docx]

*Supplementary Information*

**Electrochemical metallization cell with solid phase tunable Ge_2_Sb_2_Te_5_ electrolyte**

*Ziyang Zhang^1^, Yaoyuan Wang^1^, Guanghan Wang^1^, Jiaming Mu^2^, Mingyuan Ma^2^, Yuhan He^2^, Rongrong Yang^1^, Huanglong Li*^1^*

^1^ Department of Precision Instrument, Center for Brain Inspired Computing Research, Tsinghua University, Beijing 100084, China, ^2^ Department of Electronic Engineering, Tsinghua University, Beijing 100084, China

*Email: [li_huanglong@mail.tsinghua.edu.cn](mailto:li_huanglong@mail.tsinghua.edu.cn)

a
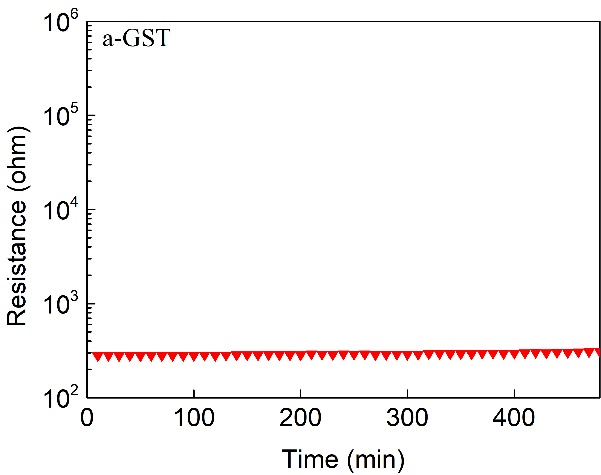
b
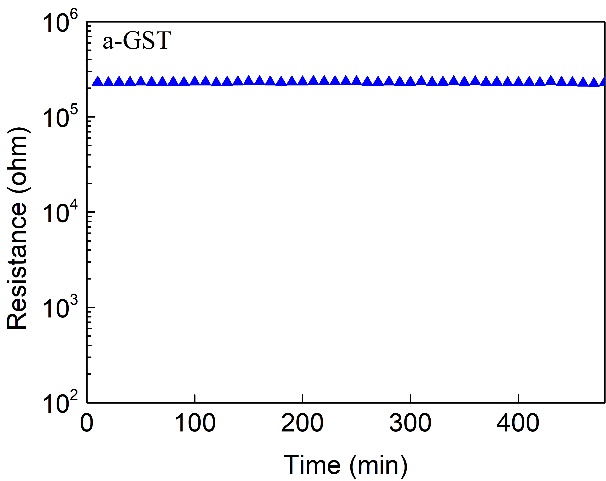


c
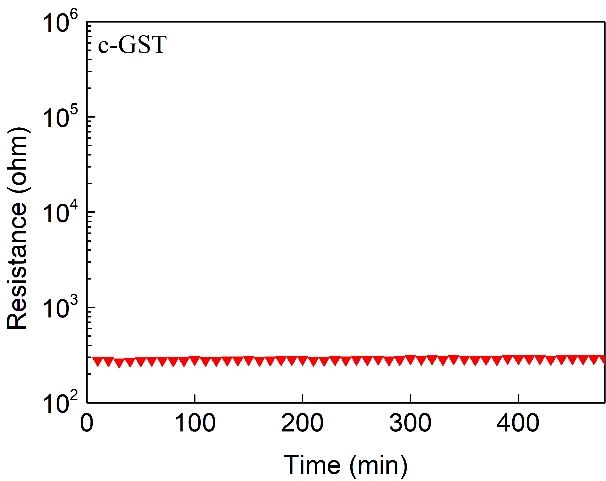
d
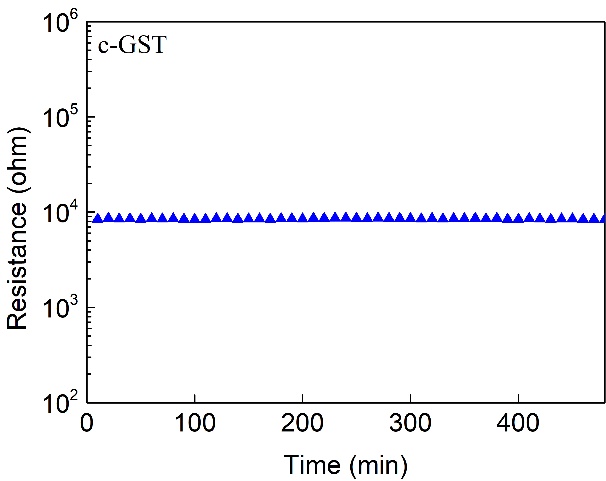


**Figure S1**. a) The eight-hour retention of the LRS for the Ag/a-GST/Pt device. b) The eight-hour retention of the HRS for the Ag/a-GST/Pt device. c) The eight-hour retention of the LRS for the Ag/c-GST/Pt device. d) The eight-hour retention of the HRS for the Ag/c-GST/Pt device.

a
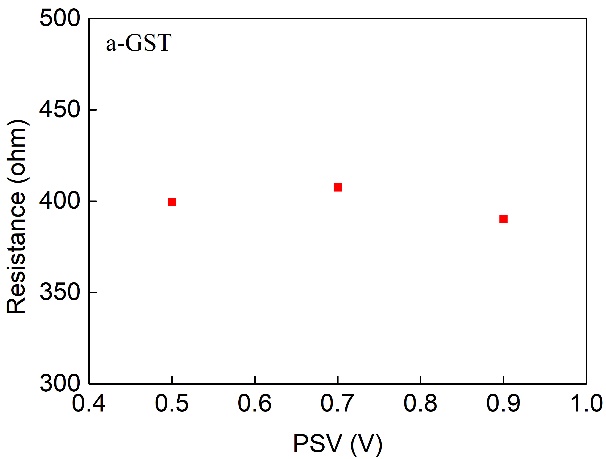
b
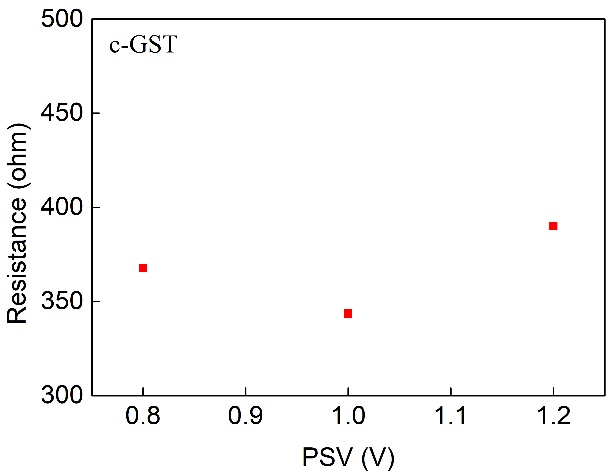


**Figure S2**. a) The LRs of the Ag/a-GST/Pt device obtained under the 0.5V, 0.7V, 0.9V PSVs. b) The LRs of the Ag/c-GST/Pt device obtained under the 0.8V, 1.0V, 1.2V PSVs. The junction area of the device is 2$\times$2 μm^2^.

a
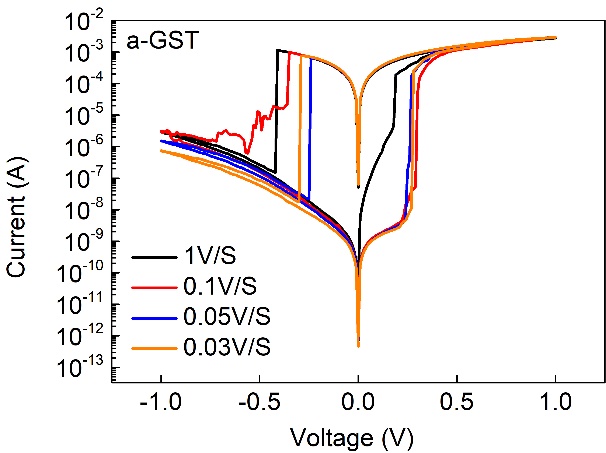


b
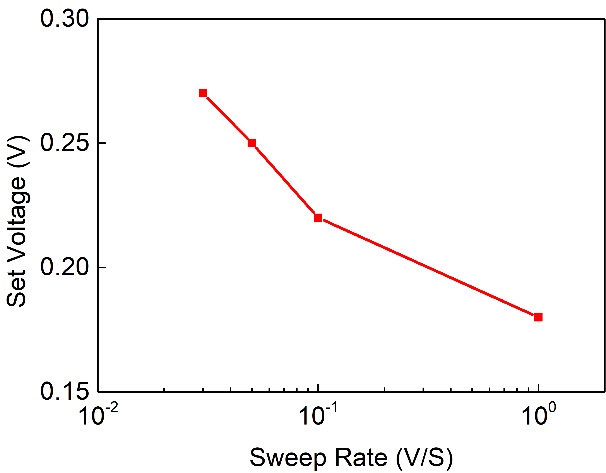
c
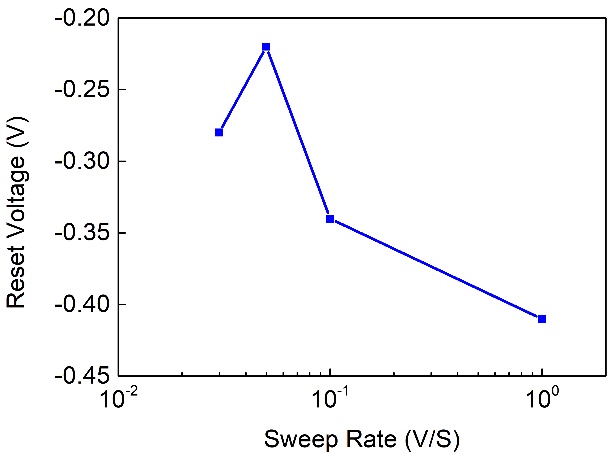


d
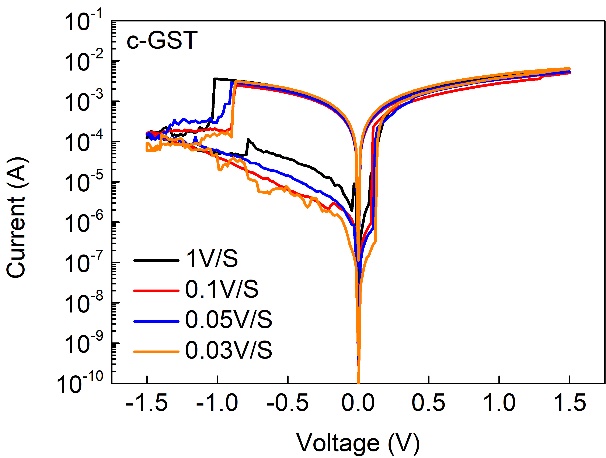


e
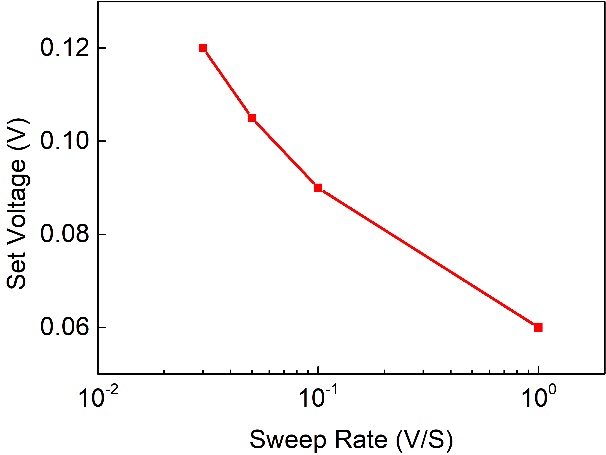
f
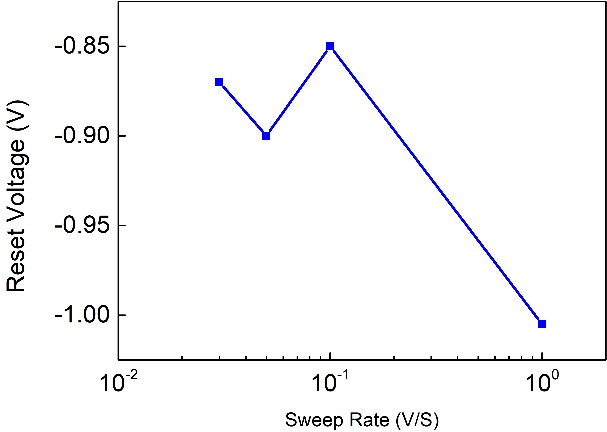


**Figure S3** a) The DC I-V curves under different sweep rates for the Ag/a-GST/Pt cell. The PSVs are fixed to 1 V. b) The influence of the sweep rate on the set voltage. c) The influence of the sweep rate on the reset voltage. d) The DC I-V curves under different sweep rates for the Ag/c-GST/Pt cell. The PSVs are fixed to 1.5 V. e) The influence of the sweep rate on the set voltage. f) The influence of the sweep rate on the reset voltage. The junction area of the device is 2$\times$2 μm^2^.

a
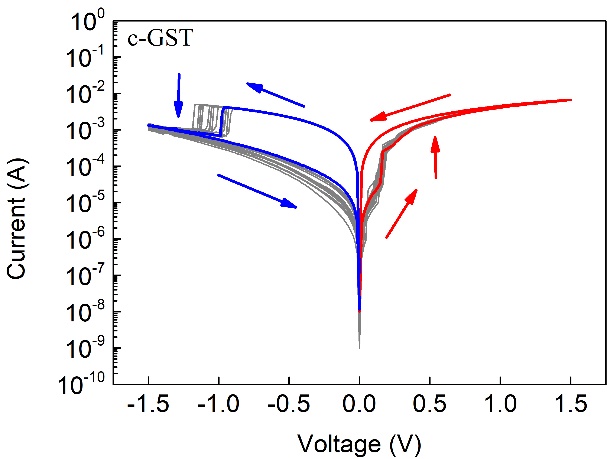
b
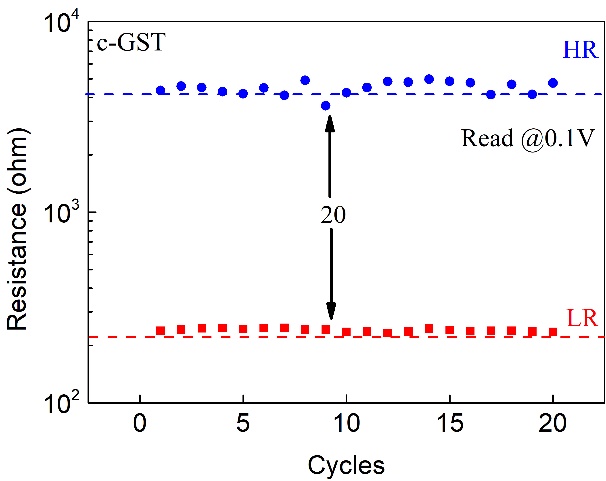


**Figure S4** a) The DC I-V curves of 20 consecutive sweep cycles for the control Ag/c-GST/Pt cell fabricated by annealing the Pt/GST layers before the Ag top electrode is deposited. b) The LR and HR of the control Ag/c-GST/Pt cell for 20 consecutive sweep cycles. The voltage of the read pulse is 100 mV. The junction area of the device is 2$\times$2 μm^2^.

We fabricate a control device (A) where the Ag top electrode is deposited after the Pt/GST layers have been annealed at 220 degree for 15 minutes. The DC I-V curves of A device are shown in figure S4. It can be seen that A device has the smallest SET voltage around +0.1V and the largest RESET voltage around -1.0V compared with those of its as-deposited amorphous GST-based device counterpart (B, see figure 2) and its crystalline GST-based device counterpart (C, see figure 4) which is annealed after the Ag top electrode has been deposited.

For device C, it is expected that unintentional out-diffusion of Ag into the GST may occur during annealing. It is generally believed that for electrolyte with high initial mobile cations the SET switching will take place at low voltage. However, the contrary is found here. To reconcile the contradiction, we propose a possible origin of the larger SET voltage and smaller RESET voltage of the device C that is annealed after the silver has been deposited than those of the device A. During the annealing of the Pt/GST/Ag stack, Ag_x_Te could be formed at the interface of GST: Ag, becoming a new cation supplier for the ECM cell. Ag_x_Te based ECM devices have be reported to have larger SET voltage due to the higher energy for silver ionization than its pure Ag counterpart. ^[1, 2]^ On the other hand, the smaller RESET voltage of Ag_x_Te based ECM devices has been attributed to the good ionic conductivity of Ag_x_Te that allows for the efficient reset because of a more efficient flux of ions out of the switching layer back into the supply layer during reset.^[2]^ We are interested in carrying out systematic investigations on the effects of annealing induced Ag out-diffusion and the possible formation of additional interfacial layer, such as Ag_x_Te, in the near future on the basis of the current work.

By comparing the switching behavior of device A, B and C, the impact of the phase transition of GST can be decoupled from the Ag related processes. The amorphous to crystalline phase transition of GST tends to increase the RESET voltage and decrease the SET voltage of the device. The possible Ag related effects induced by annealing, however, have the opposing tendency but are not strong enough to fully compensate the effect of the phase transition of GST; in other words, GST remains to be a key factor in tuning the device properties. Nevertheless, in the real operation of the device, Pt/GST/Ag stack will still be subject to any possible thermal effect, such as Joule heating, as a whole.

**Reference**

[1] J. Yoo, J. Song and H. Hwang, Nanotechnology **2018**, 29, 36.

[2] W. Devulder, K. Opsomer, J. Meersschaut, D. Deduytsche, M. Jurczak, L. Goux, and C. Detavernier, ACS Comb. Sci. **2015**, 17, 334-340.
